# Does gamification increase engagement with online programs? A systematic review

**DOI:** 10.1371/journal.pone.0173403

**Published:** 2017-03-31

**Authors:** Jemma Looyestyn, Jocelyn Kernot, Kobie Boshoff, Jillian Ryan, Sarah Edney, Carol Maher

**Affiliations:** 1 School of Health Sciences, University of South Australia, Adelaide, South Australia, Australia; 2 Alliance for Research in Exercise, Nutrition and Activity (ARENA), School of Health Sciences & Sansom Institute for Health Research, University of South Australia, Adelaide, South Australia, Australia; Universite Toulouse 1 Capitole, FRANCE

## Abstract

**Background:**

Engagement in online programs is difficult to maintain. Gamification is the recent trend that offers to increase engagement through the inclusion of game-like features like points and badges, in non-game contexts. This review will answer the following question, ‘Are gamification strategies effective in increasing engagement in online programs?’

**Method:**

Eight databases (Web of Science, PsycINFO, Medline, INSPEC, ERIC, Cochrane Library, Business Source Complete and ACM Digital Library) were searched from 2010 to the 28^th^ of October 2015 using a comprehensive search strategy. Eligibility criteria was based on the PICOS format, where “population” included adults, “intervention” involved an online program or smart phone application that included at least one gamification feature. “Comparator” was a control group, “outcomes” included engagement and “downstream” outcomes which occurred as a result of engagement; and “study design” included experimental studies from peer-reviewed sources. Effect sizes (Cohens d and 95% confidence intervals) were also calculated.

**Results:**

1017 studies were identified from database searches following the removal of duplicates, of which 15 met the inclusion criteria. The studies involved a total of 10,499 participants, and were commonly undertaken in tertiary education contexts. Engagement metrics included time spent (n = 5), volume of contributions (n = 11) and occasions visited to the software (n = 4); as well as downstream behaviours such as performance (n = 4) and healthy behaviours (n = 1). Effect sizes typically ranged from medium to large in direct engagement and downstream behaviours, with 12 out of 15 studies finding positive significant effects in favour of gamification.

**Conclusion:**

Gamification is effective in increasing engagement in online programs. Key recommendations for future research into gamification are provided. In particular, rigorous study designs are required to fully examine gamification’s effects and determine how to best achieve sustained engagement.

## Introduction

The Internet is engrained in modern life, with the average adult spending an estimated 20 hours per week online [1]. For consumers, online products and programs deliver access to services in an easy-to-use format. However, online programs can only be effective when users demonstrate engagement over the short and/or long term.

Online engagement covers a broad range of areas, and meaningful definitions vary according to context and desired goals. Some online programs are designed to sustain user engagement for one use only (i.e. the completion of an online survey) [2], whereas others aim for more frequent engagement over a sustained period of time (i.e. health behaviour programs or online educational courses) [3]. In contrast, other studies are concerned not with time but with quality of user engagement. This is particularly evident in market research studies where researchers aim to increase quality of answers in online surveys [2], or in educational studies where researchers want to increase academic performance in online courses [4].

Research shows that individuals are more likely to remain engaged in an activity if they find it enjoyable and/or of value [5]. Gamification is one method that has been linked to increased enjoyment and engagement [6]. Gamification is a concept that first emerged in a computer games context in 2002 [7], and became more widely known from about 2010 [8]. Since that time, gamification has become more prevalent within research literature albeit with varying interpretations. The two most commonly accepted definitions are: 1) ‘The process of game-thinking and game mechanics to engage users and solve problems’ [9] and 2) ‘The use of game design elements in non-game contexts’ [10]. This term is not to be confused with ‘serious game’, which can be defined as ‘digital games used for purposes other than mere entertainment’ [11].

Consistent with descriptions provided by Deterding and colleagues [10], our use of the terms ‘gamification’ or ‘gamified applications’ refers to software that incorporates elements of games. Points, badges and leader boards are the most common gamification elements. Others include: providing clear goals, challenges, levels, progress, feedback, rewards, and stories or themes [12]. There are differences in the way that similar gamification features are described (for example badges versus rewards; challenges versus quests) and what should be classed as gamification or a serious game (for example, the use of avatars). Although such inconsistencies exist, all gamification features aim to have an effect on users’ motivation, which in turn promotes better user experience and engagement [13]. Such gamification features have been applied to a variety of settings including education, health and business [8].

Common examples of smart phone applications that attribute success to gamification include Foursquare and Nike+ [2]. Foursquare uses badges to reward users when they visit physical locations [14], whereas, Nike+ awards progress and points on completion of physical activity tasks [15].

Many opinions exist as to why gamification can be successful. For example, Xu [16] suggests that gamification can facilitate extrinsic motivation (i.e, external rewards like badges and points), which can motivate engagement in the short term. By contrast, Banfield and Wilkerson [17] suggest that competition—either with one’s self or with others—explains gamification’s success.

Opinions on the effectiveness of gamification have been mixed to date, and it is not clear yet how best it should be applied. A narrative review of 24 studies addressing the question ‘does gamification work?’ in relation to motivational affordances [6] lead to the conclusion that effectiveness of gamification may depend on the context being gamified and the qualities of the user [6]. However, there were limitations to this review, including the inclusion of studies with a small sample size and/or studies that did not have a control condition [6]. Further, the methodological quality of the studies was not assessed.

A systematic approach for identifying and appraising the literature is required to identify if gamification strategies are effective in increasing engagement in online programs. This review will address the question: are gamification features effective in increasing engagement in online programs?

## Methods

This review was undertaken and reported according to the Preferred Reporting Items for Systematic Reviews and Meta-Analyses (PRISMA) guidelines (refer to S1 Appendix) [18]

### Eligibility criteria

#### Population

To be included, the studies needed to target adults. Studies which targeted tertiary education students were included, whereas those with participants from primary or high school were excluded.

#### Intervention

Studies were included if they reported an intervention delivered entirely online, through web-based or smart phone applications, and included at least one of the following gamification features: goal setting, challenges, levels, points, progress, feedback, rewards, badges, leader boards, stories or themes. Studies that reported serious games or were focused on crowdsourcing were excluded. The full range of intervention contexts were included (e.g. health, education, marketing, computer science).

#### Control or comparator

Studies were included that reported a comparison condition specific to gamification (i.e. the control group needed to involve an alternative intervention that was identical to the intervention group aside from the gamification feature). Studies that reported a pre-post design were excluded.

#### Outcomes

To be included, the gamification intervention had to target participant engagement. Additionally, studies were included if they reported downstream outcomes (i.e. outcomes that may be expected to change as a result of increased program engagement, e.g. academic achievement in an educational program, or physical activity behaviour in a physical activity program). In order to be included, studies needed to report sufficient data for Cohen’s d effect sizes to be calculated.

#### Study design

To be included, studies had to include a control group. Both random and non-randomised designs were eligible. In addition, the studies needed to be full-length reports published in peer-reviewed sources. Both peer-reviewed journals and peer-reviewed full conference papers were included. Conference abstracts were excluded on the basis that they contained insufficient detail to assess risk of bias.

During the scoping stages, it became clear that studies were highly heterogeneous, which would preclude meta-analysis. In addition, a large number of studies were identified with very small sample sizes and low quality research designs (e.g. case studies). Therefore, we applied minimum sample size limits. Power calculations were undertaken, which suggested that studies with a sample of less than n = 54 would have poor power to detect even a large effect size (i.e, Cohen’s d = 0.8) with 80%, assuming an alpha of 0.05. Therefore, we excluded studies with a sample size n < 54.

### Information sources and search strategy

Due to the broad eligibility criteria in terms of the target population, intervention and outcomes, a comprehensive search strategy was required. In consultation with an academic librarian, broad search terms were used in order to capture as many eligible studies as possible. In addition, a broad range of electronic databases were searched, representing a wide variety of academic disciplines. To achieve these goals, eight electronic databases were included: Web of Science, PsycINFO, Medline, INSPEC, ERIC, Cochrane Library, Business Source Complete and ACM Digital Library. Following several scoping searches, it was decided that a single search term would be used in each database: Gamif*. The search was limited to English language, humans, peer-reviewed and the year of publication from 2010 to present, with the final search conducted on the 28^th^ of October 2015. In addition to electronic database searches, the reference lists from relevant articles were hand searched. Once all eligible studies were identified using these processes, the list was sent to experts in the field who were asked to identify further eligible studies.

### Study selection

Studies were screened for eligibility by two independent reviewers, with results compared and discrepancies discussed until consensus was reached. Studies were first screened based on title and abstract. Where eligibility was unclear or the abstract unavailable, the full text was obtained and reviewed.

### Data collection process and data items

Data extraction was conducted using a standardised form developed specifically for this review. For each included study, pairs of reviewers independently extracted data (JL/KB, JK/SE and CM/JR). Data related to sample size, participant characteristics (age, gender and population), recruitment method, details on intervention (gamification features used, type of software, group conditions) study design and duration of follow up, and the outcomes reported.

### Risk of bias in individual studies

The included studies varied greatly in report style and study design, making the selection of a critical appraisal tool difficult. After trialling different tools and extensive consideration by the research team, a tool was specifically created for this review based on the TREND statement for non-randomised controlled trials [19]. The tool consisted of 22 items, with items scored as 1, 0.5 or 0 based on whether the study completely satisfied, partially satisfied or did not satisfy the criteria; the higher the score the lower the risk of methodological bias. The scoring process was completed independently by pairs of reviewers (JL/KB, JK/SE, CM/JR) with any inconsistencies resolved through discussion.

### Summary measures and synthesis of results

The primary measure was engagement with the online program. The secondary measure was downstream outcomes produced as a result of engagement: for instance, knowledge, academic performance, and healthy behaviour. To enable comparison across the included studies, effect sizes (Cohen’s d and 95% confidence intervals) were calculated. Where insufficient data were included to allow effect sizes to be calculated, the individual study authors were contacted for additional information. The magnitude of the effect sizes were classified as the following; ≥-0.15 and <0.15 “negligible”, ≥0.15 and <0.40 “small”, ≥0.40 and <0.75 “medium”, ≥0.75 and <1.10 “large”, ≥1.10 and <1.45 = “very large”, and ≥1.45 “huge” [20].

## Results

### Study selection

**Fig 1:**
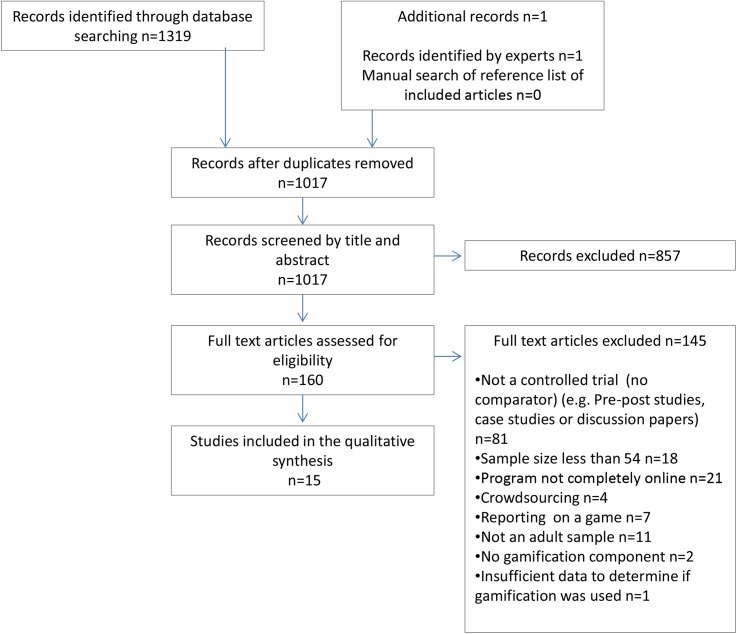
Adapted PRISMA [18] flowchart indicating flow of studies throughout the review process.

A total of 1017 studies were identified from the database search following the removal of duplicates. The flow of studies through this review is shown in Fig 1. Fifteen articles reported data on the effectiveness of gamification on engagement in online programs and were therefore included in this review.

### Study characteristics

A summary of the key characteristics of the included studies is presented in Table 1. The publication dates spanned from 2012 to 2015. Six studies were randomised controlled trials (RCT) [3,21–25]. The remaining studies (n = 9) were non-randomised controlled trials involving at least one comparator group.

**Table 1:** Summary of study characteristics.

Study	Aim	Design/ participants/ setting	Intervention	Gamification features used	Outcome measures
Allam et al. [3]	To determine the effects of gamification and social support in an educational website on physical activity, health care utilisation and correct medication use.	**Participants:** n = 155	**Groups:** a) Information only website,	Points	Physical activity (Exercise Behaviours Scale)
		**Age:** mean 58 (12)	b) information website plus online social support,	Badges	Health care utilisation (Health Care Utilization Scale)
		**%female:** 46	c) information website plus gamification features,	Reward	Prescription medication overuse (Prescription Opioid Misuse Index)
		**Recruitment:** Brochures left with health care providers	d) information website, social support and gamification and	Leader board	
		**Setting:** Patients with rheumatoid arthritis	e) control group—usual care.		
		**Context:** Health	**Duration:** 4 months		
		**Country:** Switzerland	**Follow-up post base line:** 2 and 4 months.		
		**Design:** RCT			
		**Engagement level:** Sustained			
Cechanowicz et al. [32]	To determine the effects of gamification on respondent motivation through three different types of market research surveys.	**Participants:** n = 644	**Groups:** a) Plain survey design,	Theme	Number of questions completed
		**Age:** Adults age not specified	b) partial gamification survey design and	Reward	
		**%female:** 62	c) full gamification survey design.	Challenge	
		**Recruitment:** Volunteers who regularly participate in market research surveys.	**Duration:** Single sitting	Progress elements	Number of correct answers
		**Setting:** Market research	**Follow-up post base line:** NA		
		**Context:** Marketing			
		**Country:** Canada			
		**Design:** Mixed factorial			
		**Engagement level:** Once off			
Denny [21]	To determine the effects of badges on student achievement and engagement in an online learning tool.	**Participants:** n = 1031	**Groups:** a) Educational website with badges and	Badges	Number of questions authored
		**Age:** Not reported			
		**%female:** 65			
		**Recruitment:** Students enrolled in undergraduate course	b) educational website without badges.		Number of questions answered
		**Setting:** Tertiary education			
		**Context:** Education			
		**Country:** New Zealand	**Duration:** 26 days		Number of days spent on learning tool
		**Design:** RCT	**Follow-up post base line:** 26 days		
		**Engagement level:** Sustained			
Downes-Le Guin et al. [27]	To determine the effects of gamification, decoratively visual (images and colour), functionally visual (flashing objects) and text only online survey designs on engagement.	**Participants:** n = 1007	**Groups:** a) Gamified survey,	Themes	Survey completion rate
		**Age:** Not reported	b) functionally visual survey,		
		**%female:** Not reported	c) decoratively visual survey and		
		**Recruitment:** Volunteers who regularly participate in market research surveys.	d) text only survey	Rewards	
		**Setting:** Online survey	**Duration:** Single sitting		
		**Context:** Marketing	**Follow-up post base line:** NA		
		**Country:** United States		Avatar	
		**Design:** Controlled trial			
		**Engagement level:** Once off			
Hamari [29]	To determine the effects of badges on user activity in an online sharing economy service.	**Participants:** n = 2989	**Groups:** a) Website with badges,	Badges	Number of trade proposals
		**Age:** Not reported	b) same website without badges		Number of accepted transactions
		**%female:** Not reported	**Duration:** Two years		Number of comments posted
		**Recruitment:** Existing users of the website	**Follow-up post baseline:** Not reported		Number of page views
		**Setting:** Tertiary education			
		**Context:** Community			
		**Country:** Finland			
		**Design:** Non-randomised Controlled trial			
		**Engagement level:** Sustained			
Hamari [28]	To determine the effect of badges on user retention, usage activity and social interaction in an online trading service.	**Participants:** n = 3234	**Groups:** a) Control- received badges only,	Badges	Number of trade proposals
		**Age:** Not reported	b) social comparison component,		Number of accepted transactions
		**%female:** Not reported	c) clear goal component and		Number of comments posted
		**Recruitment:** Existing users of trading service	d) both social comparison and clear goal components	Social comparison (leader board)	Number of page views
		**Setting:** Tertiary education	**Duration:** 1.5 years		
		**Context:** Community	**Follow-up post base line:** Not reported		
		**Country:** Finland		Goals	
		**Design:** 2x2 field experiment			
		**Engagement level:** Sustained			
Harms et al. [13]	To determine the effect of badges on user experience and response behaviour for an online survey.	**Participants:** n = 139	**Groups:** a) Gamified survey and	Badges	Time spent in survey
		**Age:** Adults age not specified	b) control- conventional survey		
		**%female:** 37	**Duration:** Single sitting		
		**Recruitment:** Through email and Facebook	**Follow-up post base line:** NA		Completion of survey
		**Setting:** Remote			
		**Context:** Marketing			
		**Country:** Austria			
		**Design:** Controlled trial			
		**Engagement level:** Once off			
Jang et al. [30]	To determine the effects of gamification on learning in a web-based learning environment.	**Participants:** n = 114	**Groups:** a) Gamified system,	Points	Time taken to complete quizzes
		**Age:** mean 21	b) gamified system with time pressure and	Levels	
		**%female:** 35	c) control- only learning content and quizzes (no gamification)	Avatar	
		**Recruitment:** Student volunteers	**Duration:** Not reported	Challenges	Number of completed quizzes
		**Setting:** Tertiary education	**Follow-up post base line:** Not reported		
		**Context:** Education			
		**Country:** South Korea			Accuracy of quizzes
		**Design:** Controlled trial			
		**Engagement level:** Sustained			
Juzwin et al. [33]	To determine the effects of gamification on engagement with an online evaluation platform.	**Participants:** n = 79	**Groups:** a) Control group- standard evaluation method,	Quests	Accuracy of web page ratings
		**Age:** Adult age not specified	b) same method with bets and		
		**%female:** Not reported	c) same method with quests, bets and quizzes.		
		**Recruitment:** Asked a group of students	**Duration:** One month	Bets	
		**Setting:** Tertiary education	**Follow-up post base line:** Not reported		
		**Context:** Marketing			
		**Country:** Poland		Quizzes	
		**Design:** Controlled trial			
		**Engagement level:** Sustained			
Krause et al. [26]	To determine the effects of gamification on retention and learning achievement on a Massive Open Online Course (MOOC).	**Participants:** n = 206	**Groups:** a) Gamified version,	Achievements	Number of videos watched
		**Age:** Adult age not specified	b) gamified version with social elements and	Badges	
		**%female:** 42	c) control- access to MOOC with no gamification.	Points	
		**Recruitment:** Promoted by lecturers	**Duration:** Not reported	Leader board	Test performance (Exam)
		**Setting:** Tertiary education	**Follow-up post base line:** Not reported	Challenge	
		**Context:** Education		Avatars	
		**Country:** Germany			Quiz accuracy
		**Design:** Controlled trial			
		**Engagement level:** Sustained			
Landers [22]	To determine the effects of gamification on engagement and academic outcomes in an online psychology course.	**Participants:** n = 86	**Groups:** a) Educational website with leader board and	Leader board	Number of edits made to website
		**Age:** Mean 32	b) non-gamified educational website		
		**%female:** 75	**Duration:** Ten weeks		
		**Recruitment:** Students enrolled online Psychology course	**Follow-up post base line:** Not reported		
		**Setting:** Tertiary education			
		**Context:** Education			
		**Country:** USA			
		**Design:** RCT			
		**Engagement level:** Sustained			
Mekler et al. [23]	To determine the effects of points, leader boards and levels on user behaviour in an online image annotation task.	**Participants:** n = 295	**Groups:** a) Control- no gamification,	Points	Number of tags generated
		**Age:** Mean 33 (12)	b) access to points,		
		**%female:** 65	c) access to levels and		
		**Recruitment:** Volunteers on a study register	d) access to leader boards	Leader boards	
		**Setting:** Remote	**Duration:** Not reported		
		**Context:** Academic	**Follow-up post base line:** NA		
		**Country:** Switzerland		Levels	
		**Design:** RCT			
		**Engagement level:** Once off			
Mekler et al. [24]	To determine the effects of points and meaningful framing (providing meaning to tasks) on user performance and motivation.	**Participants:** n = 172	**Groups:** a) Points only,	Points	Number of tags generated
		**Age:** Mean 33	b) meaningful framing only and		
		**%female:** 72	c) access to both points and meaningful framing,		
		**Recruitment:** Not reported	d) control- no points or meaningful framing		
		**Setting:** Remote	**Duration:** Single sitting		
		**Context:** Psychology	**Follow-up post base line:** NA		
		**Country:** Switzerland			
		**Design:** RCT			
		**Engagement level:** Once off			
Monterrat et al. [31]	To determine the effects of gamification features that match users’ profiles on engagement with an educational website.	**Participants:** n = 280	**Groups:** a) Educational website with two gamification features that best matched participant profiles and	Leader board	Time spent on the platform
		**Age:** Adult age not specified	b) educational website with two gamification features that ‘worst’ matched profiles	Reward	
		**%female:** Not reported	**Duration:** Three weeks	Feedback	
		**Recruitment:** Not reported	**Follow-up post base line:** Three weeks	Progress elements	
		**Setting:** Not reported		Challenge	
		**Context:** Education			
		**Country:** France			
		**Design:** Controlled trial			
		**Engagement level:** Sustained			
Morschheuser et al. [25]	To determine the effects of gamification on engagement with a corporate educational intranet.	**Participants:** n = 68	**Group:** a) Educational materials with points and leader boards and	Points	Time spent
		**Age:** Range 17–55	b) educational materials without gamification.		
		**%female:** Not reported	**Duration:** 30 days		
		**Recruitment:** Major banks	**Follow-up post base line:** NA	Leader boards	Number of articles read
		**Setting:** Corporate			
		**Context:** Business			
		**Country:** Switzerland and Germany			Number of questions answered
		**Design:** RCT			
		**Engagement level:** Once off			

#### Population

Studies varied greatly in regards to where they were conducted; with the most prominent being Switzerland (n = 4) [3,23–25], followed by Germany (n = 2) [25,26], the United States of America (n = 2) [22,27] and Finland (n = 2) [28,29]. Other countries included Canada, New Zealand, Austria, South Korea, Poland, France and India.

The total number of participants across all 15 studies was 10,499. The majority of studies were conducted in a tertiary educational context (n = 6) [21–23,26,30,31], followed by market research (n = 4) [13,27,32,33] community (n = 2) [28,29], psychology (n = 1) [24], corporate (n = 1) [25] and health (n = 1) [3].

#### Gamification features

The most common gamification features evaluated were leader boards [3,22,23,25,26,28,31], badges [3,13,21,26,28,29], points [3,23–26,30] and rewards [3,26,27,31,32]. Six studies evaluated a single gamification feature [13,21–23,29], while nine examined the combined impact of multiple gamification features [3,25–28,30–33].

#### Level of engagement

Of the 15 studies reviewed, six examined engagement with online programs in a single sitting [13,23–25,27,32], while the remaining nine studies examined engagement over a sustained period [3,21,22,26,28–31,33], ranging from three weeks to one year. In some instances, the length of engagement was not clear [13,23,24,26,27,30].

#### Nature of the comparison groups

The majority of studies used a control condition (n = 13), in which participants received traditional methods with no gamification [3,13,21–27,29,30,32,33]. In two studies, the comparison group included some gamification elements, which were different to the gamification features offered to the main intervention group [23,28].

#### Outcomes

The majority of studies reported outcomes directly related to engagement, such as the amount of time participants spent on the online program [13,23–25,27,30–33], ‘volume’ related metrics, such as the total number of posts contributed by a participant (i.e. number of questions completed on an online quiz or the number of comments posted to a website) [13,21–26,29,30,32,33] and the total number of views per participant [3,21,28,29]. Some studies measured ‘downstream’ outcomes (i.e. outcomes that may be expected to change, as a result of increased program engagement). Examples include performance on online questionnaires [22–26,30,32,33], physical activity [3], correct medication use [3] and health care utilisation (i.e. hospital visits or health professional appointments) [3].

### Risk of bias within studies

Table 2 summarises the risk of bias rating score for each of the included studies. Methodological quality varied widely, with scores ranging from 5 to 17.5 out of a maximum possible of 22.

**Table 2:** Risk of bias scores for the included studies. To aid in interpretability, 0 = criteria not satisfied, 0.5 = criteria partially satisfied and 1 = criteria completely satisfied.

Criteria	Item	Allam et al. [3]	Cechanowicz et al. [32]	Denny [21]	Downes-Le Guin et al. [27]	Hamari [29]	Hamari [28]	Harms et al. [13]	Jang et al. [30]	Juzwin et al. [33]	Krause et al. [26]	Landers [22]	Mekler et al. [23]	Mekler et al. [24]	Monterrat et al. [31]	Morschheuser et al. [25]
Title & abstract	1	1	0.5	0.5	0.5	0.5	1	0	0	0.5	0.5	0.5	0	0	0	0.5
Background	2	0.5	0.5	0.5	0.5	1	1	0.5	0	0.5	0.5	1	1	1	1	0.5
Participants	3	1	0.5	1	1	1	1r	1	0.5	0.5	1	1	0.5	0	0	0.5
Interventions	4	0.5	1	1	0.5	0.5	0.5	0.5	0.5	0.5	0.5	0.5	1	0.5	0.5	0.5
Objectives	5	1	1	1	1	1	1	1	0	1	1	1	1	1	1	1
Outcomes	6	1	0.5	1	0.5	1	1	1	0	0.5	1	1	0.5	0	0	0.5
Sample size	7	0.5	1	1	1	1	0.5	0	0	0.5	0.5	0.5	0.5	0	0	0
Assignment method	8	1	0.5	1	1	1	1	1	0.5	1	1	1	1	1	0	0.5
Blinding	9	0.5	0	0	0	0	0.5	0.5	0.5	0	0.5	0.5	0.5	0.5	0.5	0.5
Unit of analysis	10	1	1	1	1	1	1	1	1	1	1	1	1	1	0.5	1
Statistical methods	11	1	0.5	0.5	0.5	0.5	0.5	0.5	0.5	0.5	0.5	0.5	0.5	0.5	0	0.5
Participant flow	12	0.5	0.5	0.5	0.5	0.5	1	1	0.5	0.5	0.5	0.5	0	0	0	0.5
Recruitment	13	1	0	1	1	1	1	1	0	0	0	0	0	0	0	0
Baseline data	14	0.5	0.5	0	0.5	0	0	0.5	0	0	0.5	0.5	0	0	0	0.5
Baseline equivalence	15	1	0	0	0.5	0	0	0.5	0	0	0.5	0.5	0	0	0	0
Numbers analysed	16	1	0.5	0.5	0.5	1	0.5	1	1	0.5	0.5	0	0	0	0	0
Outcomes & estimation	17	0.5	0.5	0.5	0.5	0.5	0.5	0.5	0.5	0.5	0.5	0.5	0.5	0.5	0.5	0.5
Ancillary analyses	18	1	1	0	0	1	1	1	1	1	1	1	0	0	0	1
Adverse events	19	0	0	0	1	0	0	0	0	0	1	0	0	0	0	0
Interpretation	20	1	1	1	1	1	1	1	0.5	0	0.5	1	1	1	0	0.5
Generalisability	21	1	0	1	0	1	1	1	0	0	1	1	0	0	0	0
Overall evidence	22	1	1	1	1	1	1	1	0	0	0	1	1	1	1	1
	**Total**	17.5	12	14	14	15.5	16	15.5	7	9	14	14.5	10	8	5	10

In general, studies tended to satisfy reporting guidelines in relation to their background, objectives, allocation and unit of analysis. Few studies met reporting guidelines in relation to baseline equivalence between groups (n = 4), blinding (n = 0), adverse events (n = 2), participant recruitment (n = 6) and the statistical methods used (n = 1), with effect sizes and confidence intervals rarely being reported.

### Results of individual studies

**Fig 2:**
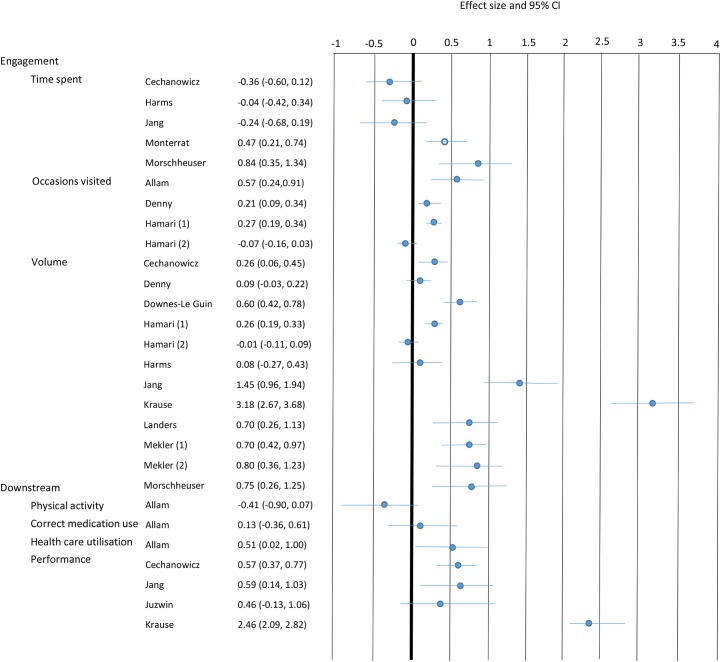
Forest plot summarising the effects of gamification on engagement and downstream behaviours. To aid interpretability, the direction of the effects is presented such that positive effects support the hypothesised effect of gamification (i.e. positive effects suggest positive impact of gamification, while negative effects suggest detrimental impact). The whiskers denote 95% confidence intervals. Where the whiskers cross zero, there is no significant effect. The unfilled circles indicate studies where both the intervention and comparison conditions included gamification.

Effect sizes (and 95% confidence intervals) were calculated for all 15 studies, and are summarised in a Forest plot in Fig 2. Meta-analysis was not undertaken due to heterogeneity between studies in terms of the application of gamification, context in which it was examined, and outcomes assessed.

### Synthesis of results

#### Engagement outcomes

Of the five studies that investigated time spent on the software as the engagement outcome and reported sufficient data to enable an effect size calculation, two studies found that gamification was associated with medium to large positive effects, while three studies found no significant effects. Four studies considered engagement in terms of number of occasions visited. Three reported significant positive effects, small to medium in magnitude, while one study showed no significant difference. Eleven studies examined engagement in terms of volume of contributions (i.e. the number of completed quizzes or number of comments made to the software); eight of these showed that gamification was associated with significant positive effects, typically medium to large in magnitude, while three studies showed no significant effect.

#### Downstream outcomes

A variety of ‘downstream’ outcomes measures were reported. Gamification was associated with significant improvement of health care utilisation, but no significant difference in physical activity or correct medication use [3].

Four studies also investigated downstream outcomes related to performance. Three of these measured performance on a knowledge test [26,30,32], and all three reported a significant positive effect associated with gamification. One study examined the impact of gamification on users’ ability to accurately rate the quality of websites and found no significant effect [33].

#### Subgroup analyses

Only one study directly compared the effectiveness of different types of gamification [23]. The effect sizes calculated for this study suggested that leader boards were more effective in increasing contributions made to their software, compared to levels (d = 0.09, 95% CI -0.24, 0.42) and points (d = 0.40, 97% CI 0.08, 0.71).

Of the eight other included studies that reported significant medium to large effects, six utilised a combination of gamification techniques [3,25–27,30,32], while two utilised single gamification techniques; these being leader board [22] and points [24]. In contrast, of the six studies that reported either no significant effect, or small significant effects, three utilised a combination of gamification techniques [28,31,33] and three utilised single gamification techniques (badges, in all cases, [13,21,29]).

The length of the intervention and the ensuing effect of gamification were also considered. Of the nine studies that reported significant medium to large effects, five studies examined engagement as measured in a single sitting [23–25,27,32], while four studies measured engagement across an extended period of time [3,22,26,30]. Conversely, of the six studies that reported either no significant effect, or small significant effects, one measured engagement in a single sitting [13], while five measured engagement across an extended period [21,28,29,31,33].

No clear pattern could be seen in relation to the effects of engagement and the quality of scientific reporting (risk of bias). For example, amongst the studies that reported significant medium to large effects, the mean risk of bias score was 11.9 out of 22 (range 7–17.5) while amongst the studies that reported no significant effect, or small significant effects, the mean risk of bias score was 12.5 (range 5–16).

## Discussion

### Key findings

Taken together, the results of this systematic review suggest that gamification can increase engagement in online programs, and enhance related outcomes, such as learning and possibly health behaviour. Most research to date has evaluated the impact of multiple gamification features used in combination. Preliminary evidence suggests that leader boards may be a particularly useful form of gamification to increase engagement. It appears that the efficacy of gamification for increasing engagement may have a time effect, with a clear positive impact in studies conducting activities in a single sitting, with results more mixed for studies examining gamification and engagement over a sustained period.

Engagement was quantified in a number of ways. Approaches included measures associated with direct engagement (such as the amount of time spent on the software), the number of occasions the software was visited and the amount of contributions made, as well as downstream outcomes that occurred as a result of engagement (including performance and physical activity). The results were generally positive for all forms of engagement.

However, the positive effect of gamification on engagement appeared to lessen over time. This result is not surprising, given that extrinsic rewards such as badges and points tend to wear off after a short period of novelty [34,35]. For instance, popular gamification app ‘Foursquare’ experienced a large reduction in engagement six to twelve months after its initial implementation [36], suggesting that gamification is more effective on engagement in the short term.

This review also provided preliminary evidence that leader boards are a particularly effective form of gamification. This is consistent with previous research indicating that social comparison promotes motivation through competition amongst peers [37]. Secondly, leader boards are more tangible and can relate more to real life. In comparison, points and badges are more arbitrary and can lack meaning, making them less effective in motivating users to engage in activities [38].

This systematic review provides a succinct snapshot of the current state of gamification and engagement science. To date, most studies have arisen from European countries. A growing number of publications have appeared each year, with just one eligible publication in 2012, through to seven publications in 2015. A variety of forms of gamification have been examined: mostly leader boards, badges, points and rewards, and commonly in combination. To date, the evaluation of gamification to increase engagement has predominantly related to tertiary education and marketing contexts. Risk of bias assessment suggests that the quality of reporting of studies is reasonably low. This may reflect the fact that this is a young field of scientific endeavour. It is important to also acknowledge that the studies included in this review came from a wide variety of academic disciplines, and reporting conventions vary between disciplines.

In general, limited detail of which gamification features were used and how they were incorporated in the online program were provided, making it difficult to determine study eligibility and the true intervention effects. The limited reporting of gamification features also impacts potential for study replication. In addition, gamification terminology varied to describe features that appeared similar between studies (“badges” versus “rewards”; “challenges” versus “quests”).

### Strengths

To our knowledge, this is the first systematic review conducted on gamification and its influence on engagement. The search strategy was very broad, allowing software created for a wide variety of contexts, populations and purposes to be included. In addition, the search was performed in a large number of databases, including databases covering a range of academic disciplines. Findings were reported in accordance with the PRISMA guidelines [18], and included rigorous and comprehensive searching, data collection and critical appraisal processes; these processes were also conducted in duplicate ensuring accuracy of the review. Finally, experts in the field were contacted to identify other eligible studies and authors were contacted to obtain additional information to improve the accuracy of reporting.

### Limitations

A key limitation of this review was that meta-analysis was not possible due to the large degree of heterogeneity between studies in terms of the target population, interventions and outcomes measured. Furthermore, it is important to recognise the possibility of reporting and publication biases. Reporting bias is possible as the search was limited to English and peer reviewed studies only: it is likely that these limits reduced the number of studies that could have been identified and potentially included in the review. As with any review, there is a possibility that studies that report unfavourable results are underreported (not published) leading to more favourable interpretations of the evidence base.

### Recommendations for future research

The following key recommendations are made for future research in this field:

1. Given the promising impact of gamification evident in this systematic review, and the narrow range of contexts in which gamification has been evaluated to date, it is recommended that further research is undertaken to explore the effectiveness of gamification on engagement in a wide variety of contexts, including health contexts.
2. Discussion amongst experts is needed to support consistent reporting of gamification features. In particular, reporting guidelines are needed detailing specifics regarding what gamification features are being used, how they are being implemented and for what reasons.
3. Given that most studies to date have examined combinations of gamification features, further research is needed to understand the impact of specific types of gamification. This review found preliminary evidence that leader boards are particularly effective; however, further research is needed to confirm this.
4. To date, strongest evidence supports gamification boosting engagement in the short term. Some studies have shown sustained benefits in the longer term, suggesting gamification has a role in supporting sustained engagement. However, further work is needed to understand how gamification is most effectively implemented to support long-term engagement.
5. More high quality, rigorously-designed studies are needed in the field of gamification. Randomised controlled trials that are directly aimed at investigating engagement with gamification are recommended.

## Conclusion

Gamification promises to increase engagement with online programs. To date, gamification has been used primarily in education and market research contexts, with reporting standards and methods of engagement varying amongst studies. Results of this systematic review indicate that gamification positively impacts engagement and downstream behaviours (e.g. health behaviours and academic performance), especially in the short term. Preliminary evidence also indicates that leader boards may be a particularly effective gamification feature, however more research is required to confirm this. More rigorous research designs are needed to determine effectiveness of gamification in different settings, and to investigate how gamification can be used to increase long-term engagement in online programs.

## Supporting information

S1 Appendix This is the completed PRISMA checklist. (DOCX)
